# The role of semantics in the success of crowdfunding projects

**DOI:** 10.1371/journal.pone.0263891

**Published:** 2022-02-11

**Authors:** Orel Babayoff, Onn Shehory

**Affiliations:** Bar-Ilan University, Ramat Gan, Israel; Jahangirnagar University, BANGLADESH

## Abstract

Crowdfunding platforms allow entrepreneurs to publish projects and raise funds for realizing them. Hence, the question of what influences projects’ fundraising success is very important. Previous studies examined various factors such as project goals and project duration that may influence the outcomes of fundraising campaigns. We present a novel model for predicting the success of crowdfunding projects in meeting their funding goals. Our model focuses on semantic features only, whose performance is comparable to that of previous models. In an additional model we developed, we examine both project metadata and project semantics, delivering a comprehensive study of factors influencing crowdfunding success. Further, we analyze a large dataset of crowdfunding project data, larger than reported in the art. Finally, we show that when combining semantics and metadata, we arrive at F1 score accuracy of 96.2%. We compare our model’s accuracy to the accuracy of previous research models by applying their methods on our dataset, and demonstrate higher accuracy of our model. In addition to our scientific contribution, we provide practical recommendations that may increase project funding success chances.

## Introduction

In recent years, crowdfunding has emerged as a popular financing mechanism that allows various types of projects to get funded. Three main parties are involved in crowdfunding: entrepreneurs (and their projects), individuals or groups who support the project, and online platforms, i.e., crowdfunding websites [1]. The latter bring together entrepreneurs and supporters to facilitate project funding and launching. There are many crowdfunding websites, of which the most popular ones are *Kickstarter* and *Indiegogo* [2]. By 2020, 34 billion dollars were raised by crowdfunding [3]. The average success rate of funded crowdfunding projects in *Kickstarter* is 37.5% [4]. In this paper, we refer to a project as a funding success (FS) if it achieved its funding goal (i.e., it raised at least 100% of the funding goal).

With the growth in popularity and scale of crowdfunding, achieving success in funding has attracted much popular and scientific attention. Studies have examined properties of successful projects, developed models of such projects, and tried to predict success based on those models. For example [5], examined the type of funding requested and its effect on funding success. In [6], the authors examined the effect of social engagement on success. Work in [7] studied the meta-data features that explain funding success, and based on the meta-data features they developed a prediction model for funding success. While models in the art rely on metadata features and topic analysis for predicting crowdfunding success, they seldom consider semantic features. As we show, this lack negatively affects prediction accuracy. In this study we aim to improve prediction accuracy. To do that, we focus on extraction and analysis of semantic aspects of project posts. In contrast to prior studies, we take a more comprehensive approach to studying and predicting funding success. Unlike prior studies, we use unique features (for example, the use of buzzwords) and perform extensive research on the semantics of the projects’ text. We use semantic features extracted from the text to build a predictive model of funding success. Further, to increase the accuracy of our results, we incorporate already known features that affect funding success. This approach leverages known results and improves upon them to provide high-quality funding success prediction. As we demonstrate, the introduction of semantics into funding success prediction indeed improves accuracy, thus justifying our method.

Projects seeking crowdfunding range from one-time events to starting up small companies. Accordingly, these projects differ in terms of the requested investment amount and the type of compensation promised to investors. There are four primary types of crowdfunding [5,8,9]:

1. Reward crowdfunding: Investors contribute in return for non-financial benefits.
2. Debt crowdfunding: Investors fund projects as lenders and are paid interest on their investment.
3. Equity crowdfunding: Investors receive a return on investment by being offered private company securities.
4. Donation crowdfunding: This type is designed for charities or those who raise money for social or charitable projects, enabling them to gather an online community to donate to a project.

Our main research objective is to find characteristics, with a focus on semantic characteristics, which affect the success of a project in meeting its funding goal via crowdfunding. Our second objective is to answer the following questions: Given a dataset of crowdfunding projects’ details as input, consisting of text such as project description, non-text metadata such as project category, funding, etc., can we predict the success of projects in meeting their funding goals? At what level of accuracy? How would diverse learning algorithms affect this accuracy? Achieving these research objectives should provide new insights into the relationship between crowdfunding project’s data and their funding success. In addition, this should provide entrepreneurs with additional and desirable ways to improve their chances of reaching their funding goals.

The study offers five main contributions. First, we demonstrate the important role of project’s semantic properties in the project meeting its fundraising goals. To this end, we perform a comprehensive semantic analysis of the project’s description, including topic analysis using LDA, word usage analysis using LIWC [10], and others.

Second, we present a novel model which is based on semantic features only, referred to as *semantic-model*, that predicts the success of crowdfunding projects in meeting their funding goals. We show that the accuracy of the *semantic-model* (in terms of F-score and correctly classified instances) is comparable to the accuracy of state-of-the-art models which are based on metadata features such as the number of updates, the number of images, etc.

Third, building upon our new insights regarding project semantics, we introduce an additional prediction model that combines semantics and project metadata, referred to as *combined-model*. The accuracy of the *combined-model* is higher than the accuracy derived in previous studies that use other models [11–14]

Fourth, we show that semantic features including *buzzwords* and LIWC are among the highly correlate features with the project’s success in fund raising.

Fifth, to the best of our knowledge, our research relies on the largest dataset used to date for developing models that predict funding success of crowdfunding projects. The dataset used included 160,125 project records from the two biggest crowdfunding websites. Even after filtering records of lesser relevance, 111,273 project records were still included in the analysis. This allows us to deliver a very accurate prediction model with very low error levels.

In addition to the scientific contributions listed above, we provide a set of recommendations that may increase project funding success chances. These recommendations are based on the results of our study and of earlier studies as well.

The semantic analysis introduced in this study is applicable beyond the specific domain examined here. Utilizing such analysis, one could study the effect of semantic properties of textual data on various target readerships. In our research, the textual data analyzed was the crowdfunding projects’ description, and the target audience was individuals and groups that support the project. Another application domain could be, e.g., one where social network posts of an organization are analyzed to examine the level of support exhibited by its followers.

The paper proceeds as follows. The Introduction section presents the state-of-the-art, the motivation for this study, and its objectives. The Background section presents background in crowdfunding as well as semantic analysis on which the study is built. The Methodology section focuses on the research methodology applied, including the acquisition of our dataset, preprocessing, feature extraction and selection. The Results Section presents the empirical evaluation of our research model and discusses the results. It further compares our models to models in earlier studies. Finally, in the Conclusion and Future Work section we summarize and discuss the main findings and point at future directions of our research.

## Background

In this section, we discuss the features used in our models, highlighting where we differ from previous studies. We also present the metric used to measure the accuracy of the models.

For semantic analysis, the projects’ textual data is required. Hence, a major data source used in this study is crowdfunding projects’ posts. Such posts typically comprise multiple elements, including (but not limited to): title, subtitle, category, FAQ, project description, video, funding goal, time to achieve the goal, money raised, contact information, updates, backers, support ways and the names of the entrepreneurs or company. Our semantic analysis including topic analysis using LDA, word usage analysis using LIWC and novel features including the use of buzzwords, feeling words and explanation words.

### Buzzwords

The analysis we performed on posts examined their use of buzzwords [15]. Although some studies examined the use of buzzwords in project texts, the relationship between funding success and buzzwords used in the description of the project was not examined. This is a novel contribution of our research.

The buzzword dataset used in this study contains words from different categories: general conversation, education, business, sales and marketing, science and technology, politics, and current affairs.

Example buzzwords from the technology domain may be big data, cloud computing, open-source, etc. In our study, the *buzzwords* feature was the percentage contribution of the words in the buzzword dataset to each input text i.e., project’s description.

### Using Linguistic Inquiry and Word Count (LIWC)

To extract features from the text, we used the Linguistic Inquiry and Word Count (LIWC) software tool. LIWC includes text analysis software along with a group of built-in dictionaries [10]. Example of dictionaries are (* denotes stems):

- Negative emotion: abuse*, sorry, fury, tears, painf*, tragic*, etc.
- Positive emotion: accept, funn*, ador*, pleasant*, glad, etc.

The LIWC analysis measures the appearance of dictionary words in a specific text. The measure is a numeric value in the range [0..1]. It reflects the degree to which the text being analyzed is related to the theme of the dictionary. Specifically, the analysis works as follows. N_T_ = the number words in the description text T and M_D_ = {*w*_*1*_,..,*w*_*n*_}, the set of *n* words in dictionary D. Accordingly, let N_*w*_ = the number of occurrences of the word *w* from M_D_ in T. S_D_ = ∑_*i = 1*..*n*_ N_*wi*_ is the sum of N_*w*_ over all words *w* in M_D_. The output of LIWC is V_D_ = S_D_/N_T_. This value is the value of the feature that corresponds to D. In practice, V_D_ measures the frequency of appearance of words (and their stems) from a specific dictionary D in the examined text.

Previous studies on crowdfunding used LIWC to analyze the textual description part of projects (though they did not look for buzzwords) and arrived at high model accuracies (e.g., F-score > 0.8) [14].

The main difference between the present research and those studies is that the number and diversity of features in our study are much larger than previously published, and our dataset is significantly greater as well. As a result, we arrive at much higher model accuracy.

### Latent Dirichlet Allocation (LDA)

Topic modeling methods are used in text mining tasks to extract topics from text. Latent Dirichlet allocation (LDA) is a widely used topic modeling [14]. LDA algorithm considers each document as a collection of topics, where each word in the document belongs to one or some of these topics. Within a topic to which it belongs, the word has a weight that expresses its importance in the context of that topic. A topic is expressed by the set of words belonging to it and their associated weights. The value of the topic will be the sum of those weights. Once the algorithm has a specified number of topics, it rearranges the distribution of the topics within the documents and the keyword distribution within the topics to obtain a good configuration of the topic–keyword distribution [14].

Previous studies on crowdfunding used LDA to perform topic analysis on the text of project updates, though on a much smaller and very specific dataset compared to our research [14]. Another study used LDA to analyze Chinese crowdfunding websites and arrived at an accuracy level (F-score) of 86.7%. In our study, we used LDA to extract features from project descriptions, where each topic is a feature. We used the Gensim Python package LDA implementation (from *github.com*). The latter is known to run faster than other implementations and it generates better topic segregation [16].

To execute LDA, we performed some preprocessing. First, we tokenized each sentence into a list of words, removing punctuation, unnecessary characters, email addresses, and names. Once this was completed, we defined functions that remove the stop words, which generate bigrams (two words frequently occurring together in the document) and perform lemmatization (the process of grouping together the inflected forms of a word so they can be analyzed as a single item).

The three main inputs to the LDA topic model are the dictionary, the corpus, and the number of topics. Model perplexity and topic coherence provide a convenient measure to judge how good a given topic model is [16]. To avoid overfitting, we varied the number of topics from 10 to 50 and chose the one that derived the highest coherence value and the smallest number of topics.

We ran the LDA on three datasets (of which details are in the Methodology section) and derived three corresponding sets of topics. For each dataset, the input to the LDA was textual descriptions of the projects in that dataset. The output was a set of topics and the percentage contribution of each topic to each input text. In our study, every topic was considered as a feature for developing the prediction model, and the percentage contribution of a topic was the value of the feature that represented that topic. There were 30 topics that derived the highest coherence for the All_D dataset, 15 for the Tech_D dataset, and 15 for the Market_D dataset.

### Metadata

Additionally, we incorporated metadata features known to affect funding success. The metadata features we used were extracted from projects’ posts via Python web scraping. The set we used for our analysis included the number of photos, the number of videos, the number of updates, the funding goal, the number of previously created projects by entrepreneur, the number of successfully completed (a.k.a. backed) entrepreneur’s projects, and the time to achieve that goal (i.e., the project’s deadline) [7,12,17–22].

### Additional semantic features

In addition to topics identified by LIWC and LDA, we explored additional topics that could have a high correlation to FS by reviewing those used by studies in the field of textual analysis. The following additional topics were deduced:

- Explanation words: example, explain, for instance, i.e., mean, in other words, in that, that is, etc. [23].
- Feelings words: angry, annoyed, afraid, awkward, affectionate, anxious, alarmed, awed, aggravated, amazed, etc. This bag of words comes from the unification of LIWC dictionaries that include feeling words [24].

### Performance metrics

We used the following metrics to measure the performance of our models. Precision is the ratio of correctly predicted positive records to the total predicted positive records. Recall is the ratio of correctly predicted positive records to the all records in actual class. F-score (also denoted F_1_) is the weighted harmonic mean of a model’s precision and recall. These metrics are calculated as follows:

$$\begin{array}{l} \mathrm{Precision}=\frac{tp}{tp+fp}\\ \mathrm{Recall}=\frac{tp}{tp+fn} \end{array}$$

$$F_{1}=(\frac{2}{{\mathrm{recall}}^{-1}+{\mathrm{precision}}^{-1}})=2\cdot \frac{\mathrm{precision}\cdot \mathrm{recall}}{\mathrm{precision}+\mathrm{recall}}.$$

Where *tp* is the number of true positive predictions. *fn* and *fp* are the number of false negatives and false positives predictions, respectively.

## Methodology

In order to meet the objectives of this study, we have developed a knowledge discovery plan. The plan comprises four main parts: obtaining and preprocessing datasets; feature selection; data analysis based on machine learning models; and, finally, evaluation of the knowledge extracted by the models.

We used a dataset of 50,000 *Kickstarter* and 50,000 *Indiegogo* projects. We obtained these data from the *Kaggle* website for 2018. Note that similarly to other studies in the field, we filtered out projects with fewer than 10 lines of description. The goal of such filtering is to avoid compromising the accuracy of the semantic analysis [14]. We also omitted from the dataset projects whose completion due date was still in the future. After filtering, we had 73,583 projects: 42,530 that achieved their funding goal (success = 1), and 31,050 projects that had not managed to achieve their funding goal (success = 0).

After filtering the dataset, we used *Beautifulsup* (a Python web scraping package) to gain additional metadata features, such as, description content, number of updates, etc. For each record in the dataset, we obtained the values of five metadata features and about 120 semantic features, including buzzwords, LIWC outputs, feelings words, explanation words, and LDA outputs. The values of the features were computed as described above. We removed LDA topics that overlapped with LIWC topics by comparing the topic sets. Whenever there was an overlap, LDA’s topic X was subsumed into LIWC’s dictionary/topic.

We preprocessed the dataset via common methods (for example, conversion of an ordinal variable to a numerical one or dataset normalization) to adjust the data to the input requirements of the data analysis algorithms we used for deriving the model.

To study the relationship between features and the project category, we built three datasets:

- All_D: comprising all projects regardless of their category.
- Tech_D: comprising only technological projects.
- Market_D: comprising only projects on marketing and business.

This division aims to address the following analysis goals. First, it facilitates improvement in the LDA topic coherence score. Second, it allows us to determine whether a category affects the features that are highly correlated with funding success. Third, to study the correlation between buzzwords and FS, it is preferable for datasets to be separated into specific sub-domains (most buzzwords we used are indeed in these sub-domains).

We utilized MANOVA (multivariate analysis of variance) to examine the similarity among the datasets. The results show that the separation by category causes statistical significance of the mean differences between the features using p-value = 0.05. We computed the Pearson correlation between all of the features we studied and the FS. All of the models and the correlation values were computed, separately, with respect to the three datasets.

**Fig 1:**
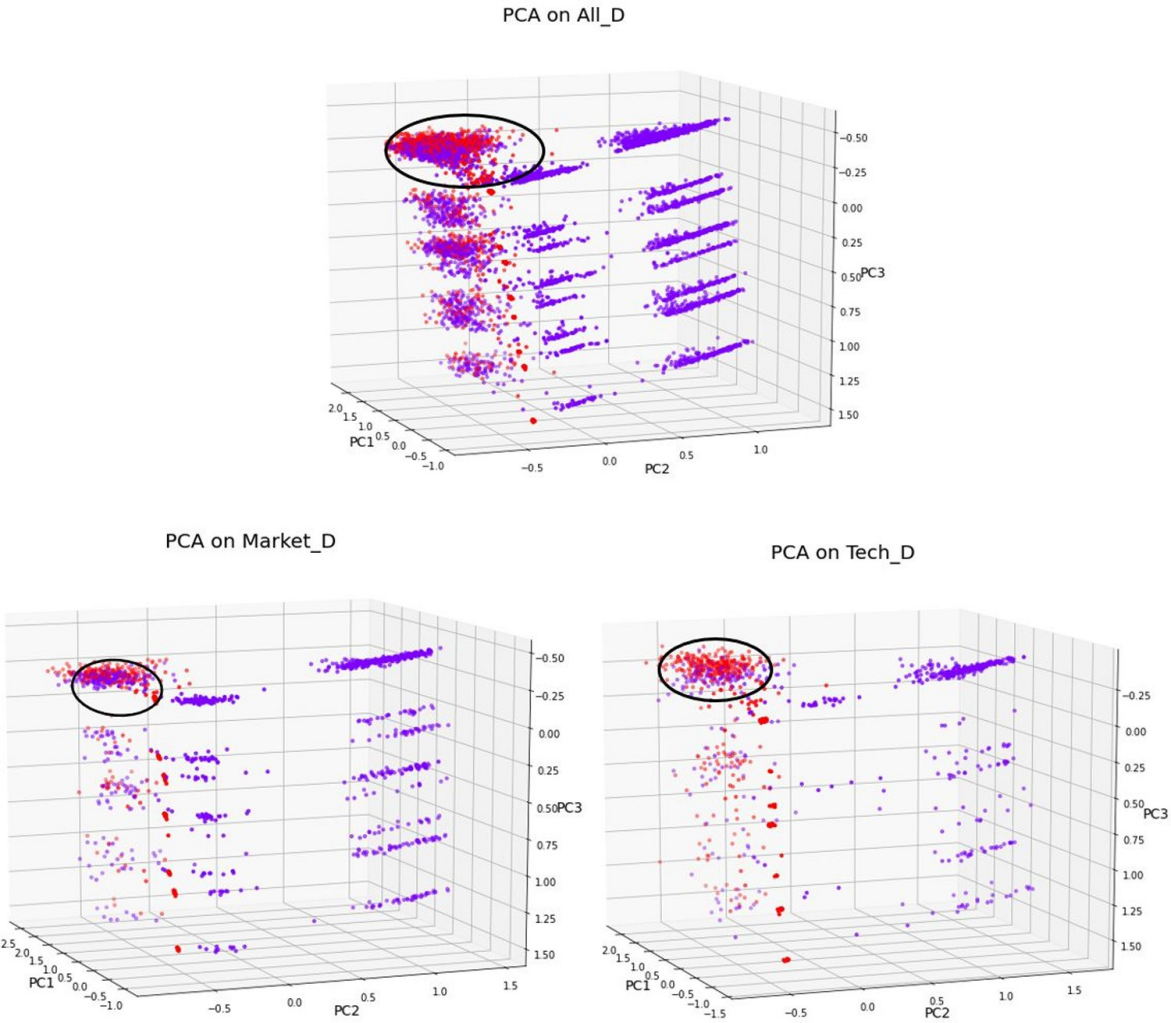
PCA on datasets.

To increase comprehensibility, we wanted to visualize the feature space and its general data structure. For this, we had to reduce the multi-dimensional space to a three-dimensional space. Thus, we utilized Principal Component Analysis (PCA) to identify the most influential features [25,26]. Following this analysis, for each dataset, the original features space was reduced into three Principal Components (PCs), as visualized in Fig 1. The sum of the explained variance ratio of these three PCs is 78% of the total variance.

The PCA plots in Fig 1 present the distribution of the All_D, Market_D and Tech_D datasets. Each dot in the plots represents a project record in the dataset. Dots in blue represent unsuccessfully funded projects and dots in red represent successfully funded projects. Most of the records of the successfully funded projects are located in the range where *PC3 < 0*.*5*, *-1 < PC2 < 0*, *1 < PC1 < 2*.

To identify the most significant set of features (MSSF) that has the highest impact on FS, we use the CFS (correlation-based feature selection) algorithm. CFS evaluates a subset of features by considering the individual predictive ability of each feature along with the degree of redundancy between them. We executed this algorithm on each dataset, separately.

In this study, as mentioned above, we developed two binary classification models to predict funding success. The labels of the two classes are *1* and *0*, where *1* is the label of the class of successfully funded projects and *0* is the label of the class of unsuccessfully funded projects. To develop those models, we utilized widely used classification algorithms (Random forest, decision trees, DNN) as well as state-of-the-art leading algorithms such as *LightGBM* [27]. The models’ design, derivation, and operational tests were conducted using the following Python packages: scikit*-learn*, *scikit-feature* and *LightGBM*.

Recall that one of our goals was to develop the *semantic-model* whose accuracy is comparable to the accuracy of existing models. To this end, we developed a model whose features are only semantic features. The inputs of that model consist of buzzwords, explanation words, feeling words and the topics derived from LIWC and LDA.

Once the *semantic-model* was developed and tested, we aimed to further improve model accuracy. To this end, we have developed the *combined-model*, whose novelty lies in the combination of semantic and meta-data features. For each of the two models, we calculated the F-score as a measure of model’s accuracy.

We employed a 10-fold cross-validation test to evaluate the prediction performance. This test has been used widely to validate the performance of models similar to ours [6,21,28]. For each fold, our data sample was randomly divided into 10 parts, then 10 experiments were performed, with nine parts used as training data for the predictive model to predict the remaining part. For each fold, we trained the LDA algorithm on the training data. The average prediction accuracy is reported.

**Fig 2:**
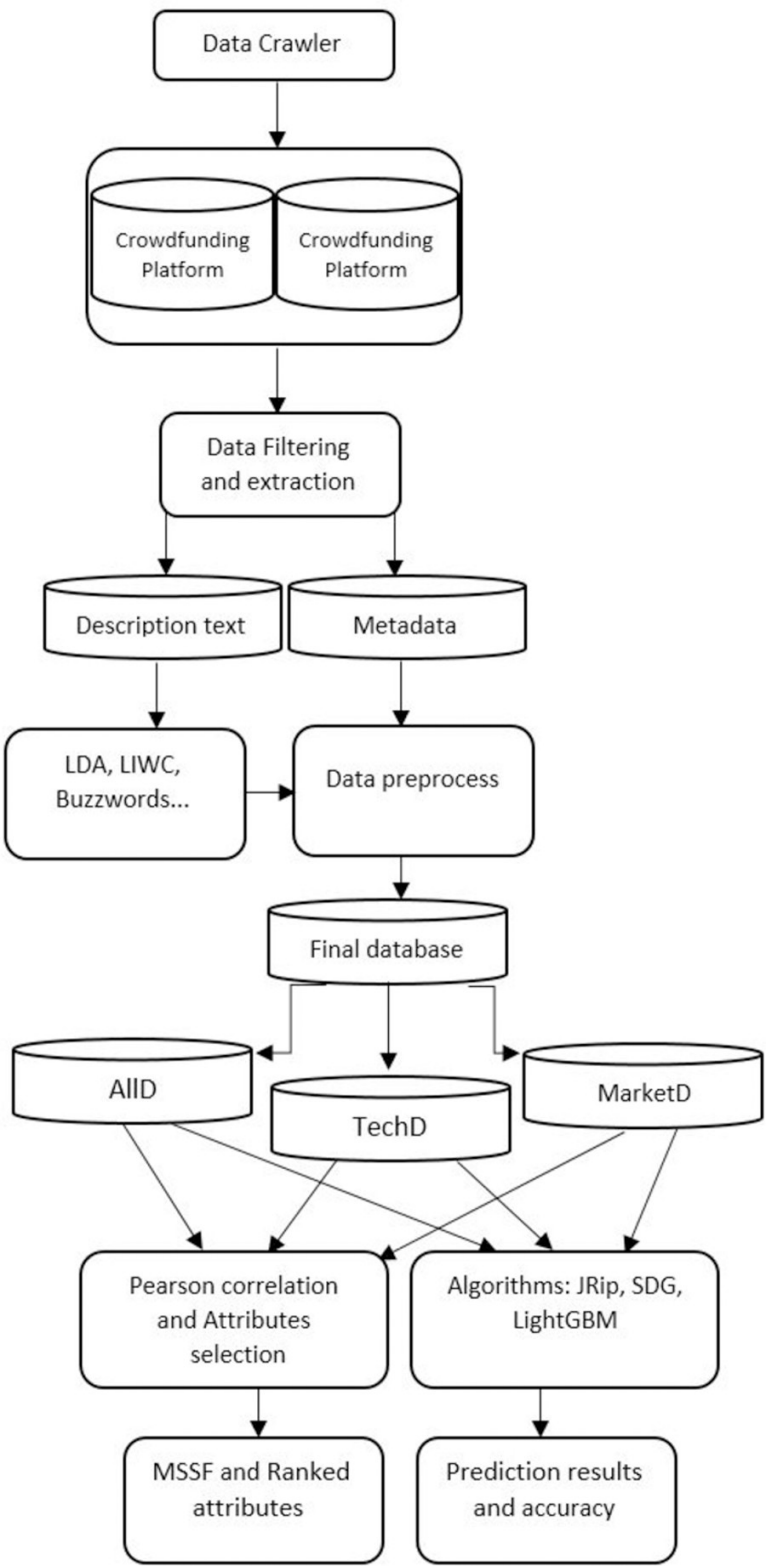
Methodology flowchart.

Our goal was to achieve an accuracy level greater than 90%. Fig 2 includes a flowchart of the methodology. To validate the results of our study, we compared our model’s accuracy to previous research models by applying their methods on our dataset.

## Results

### Feature extraction and selection

In this section, we provide details of the data setup, the usage of the LDA algorithm, the feature correlation analysis, the feature selection process, and the development of a prediction model.

#### Features that most significantly influence funding success

In what follows we describe the way in which we found the features that are most influential for FS. We used two methods. The first is the Pearson correlation between the independent features and the dependent feature (i.e., the FS). The second is the CFS algorithm.

#### Features that highly correlate with funding success

Below in Table 1, for each of the three datasets, we list the 10 features for which the highest correlation values to FS were derived. The semantics of features annotated with * are provided in LIWC documentation (R_All_D stands for Ranked features from All_D).

**Table 1:** Feature correlation with funding success.

	R_All_D	Corr.	R_Tech _D	Corr.	R_Market_D	Corr.
**1**	achieve*	0.758	achieve*	0.718	punc*	0.717
**2**	punc*	0.668	percept*	0.677	achieve*	0.696
**3**	preps*	0.625	feelings_num	0.675	preps*	0.659
**4**	feelings_num	0.599	WC*	0.663	work*	0.54
**5**	percept*	0.595	see*	0.644	see*	0.495
**6**	work*	0.592	discrep*	0.61	present *	0.494
**7**	hear*	0.583	updates	0.574	feelings_num	0.492
**8**	see*	0.582	work*	0.556	Instagram_link	0.478
**9**	discrep*	0.56	punc*	0.53	buzz_num	0.475
**10**	buzz_num	0.519	buzz_num	0.52	number*	0.468

#### Feature selection

We used the CFS algorithm to find MSSF in every dataset. The CFS algorithm evaluates subsets of features based on the individual predictive ability of each feature along with the degree of redundancy between them. The following are the algorithm’s results.

MSSF from All_D (S_ All_D):

- achieve*
- updates
- punc* (all punctuation)
- emoticon*
- funding goal
- backed (# of backed projects)
- project duration

MSSF from Tech_D (S_ Tech_D):

- backed
- funding goal
- buzz_num
- updates
- FAQ (# of FAQ in project’s page)
- project duration

MSSF from Market_D (S_ Market_D):

- funding goal
- updates
- buzz_num
- work*
- created (# of created projects)
- punc*

From the above results, we conclude that there are various semantic features that have a high correlation to FS among the features we checked based on the project’s category. Correlation values similar to those computed in our study are typical in the art [12,19]. The semantic features *buzzwords* and LIWC are among the features that have a higher correlation in all datasets. When inspecting the correlation analysis results, we can observe that among the top 10 features, about 70% of the features are the same across datasets. For example, the features *feelings_num* and *buzz_num* appear in the three top 10 lists. In contrast, the parameter *WC* is unique in the R_Tech _D top 10 list and the parameter *number* is distinctive in the R_Market_D top 10 list. An important, unique conclusion of our study is that the features correlated to FS are dependent on the project category.

### A model for predicting funding success

#### Semantic model

For the development of the *Semantic-Model*, semantic features were used as input. We utilized several machine learning algorithms (including SVM, J48, Random Forest, LightGBM, SDG, DNN, and more) on All_D. Table 2 presents the accuracies metrics of the *Semantic-Model*:

**Table 2:** *Semantic-model* performance.

Algorithm	F-score	Precision	Recall	Accuracy
LightGBM	91.1%	92.3%	89.9%	90%
SDG	89.3%	90.5%	88.1%	88.5%

#### Combined model

To develop the *combined-model*, we utilized the same classification algorithms as used for the development of the *Semantic-Model* on our three datasets, using all features. Below we list the best models (by F-score).

We used the *grid search* algorithm to optimize the hyperparameters with which each algorithm was executed (for example, for SDG we optimized batch size, number of epochs, etc.). In addition to the grid search algorithm, we utilized the *Random search* algorithm and got similar accuracies for our classification models.

The grid search algorithm selected a set of hyperparameters, and these were used for training our model. Specifically, the following hyperparameters were used for All_D dataset:

- SDG: batch size = 100, epochs = 500, learning rate = 0.01, loss function = hinge loss (SVM), lambda (regularization constant) = 0.0001.
- Random forest: number of trees = 20, max depth = infinity, max number of features = unlimited.
- LightGBM: number of leaves = 255, number of boosting iterations = 5000, Minimal number of data in one leaf = 10, learning rate = 0.01, max number of bins = 300.

As seen above, the semantic-model we have developed is comparable in accuracy with the state of the art (metadata) models, exhibiting an accuracy level of 91.2%. Further, as shown in Table 3, by combining the semantic model with the metadata model, we have achieved an accuracy level of 96.2%. Additionally, as seen across Tables 3–5, the LightGBM algorithm exhibited a high accuracy level for all three datasets. In particular, with LightGBM, the accuracy of the prediction model is greater than 94% for all datasets.

**Table 3:** All_D performance.

Algorithm	F-score	Precision	Recall	Accuracy
LightGBM	96.2%	97%	95.4%	96.2%
SDG	95.3%	95.5%	95.1%	95.1%

**Table 4:** Tech_D performance.

Algorithm	F-score	Precision	Recall	Accuracy
LightGBM	94.8%	94.8%	94.8%	94.2%
Random	93.9%	91.7%	96.2%	92.2%

**Table 5:** Market_D performance.

Algorithm	F-score	Precision	Recall	Accuracy
LightGBM	95.6%	95.9%	95.3%	95.3%
SDG	95.2%	95.4%	95%	95.4%

### Comparing our model to earlier models

To better understand the significance of our results, we compared them to results of earlier studies. To this end, we implemented two models from earlier studies and trained them on the All_D dataset to compare their accuracy to that of the *Sematic-Model* and the *Combined-Model*.

In the first study [14], developed their best model (by means of F-score accuracy metrics) and trained it on 80 LDA features that were extracted from project descriptions. Relying on their method, we tried different numbers of topics in the range of 5 to 100. We did the same for each textual corpus and observed the corresponding perplexity scores. Using perplexity, we found 30 topics to be the best fit. We denote the model that trained on those features *LDA-Model*.

In the second study [18], utilized only metadata features to develop their prediction model. We denote the model that trained on those features *Metadata-Model*.

**Fig 3:**
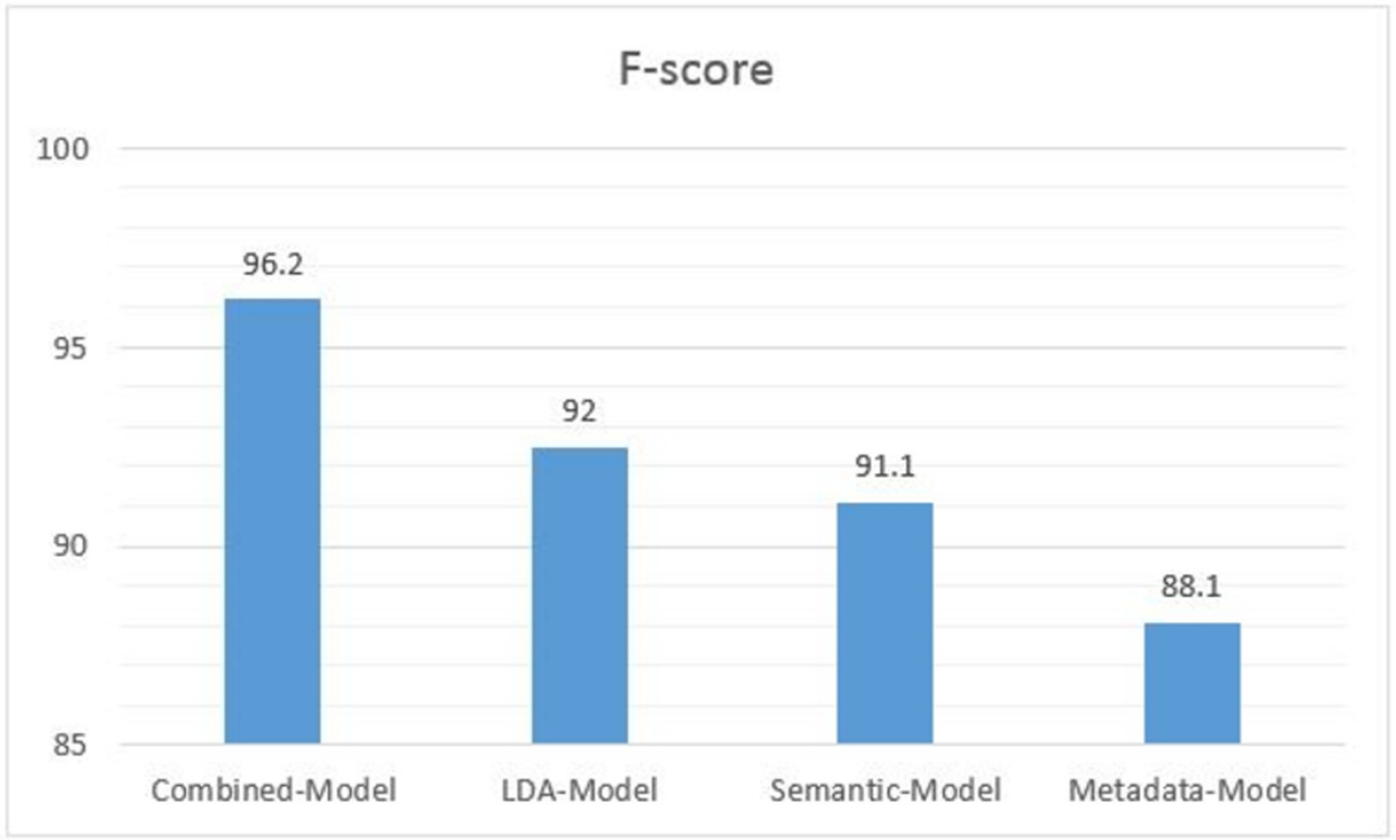
Comparison of models’ accuracy.

Our study aims to examine whether the set of features we use for prediction and the dataset on which learning was applied deliver a better model by means of F-score accuracy. To this end, we examined the influence of the learning algorithms, the feature sets and the dataset on the derived F-score. Accordingly, we trained the *LDA-Model* and the *Metadata-Model* with the algorithms that were used in the studies above, and with additional algorithms, including SVM, J48, Random forest, LightGBM, SDG, and DNN. This training was performed on All_D with 10-fold cross-validation. This extensive experimental trial led to the conclusion that neither the learning algorithms nor the dataset are a source of significant differences in F-score. The major effector of such differences is the set of features. Fig 3 presents the highest F-score metric values of the *LDA-Model*, the *Metadata-Model*, the *Semantic-Model* and the *Combined-Model*. F-score is used for consistency with the earlier studies to which we compare.

The Figure shows that the model we developed has the highest accuracy. The accuracy of the *Semantic-Model* is similar to the accuracy of the *LDA-Model* and higher than the accuracy of the *Metadata-Model*.

## Conclusion and future work

In recent years, crowdfunding platforms such as *Kickstarter* and *Indiegogo* have been offering entrepreneurs the possibility to present their projects and attract funders, and thus raise the funds necessary for their projects. The question of how different properties of the project’s presentations can increase the chance of successful project funding is important. While previous studies have identified some metadata features and topic analysis for predicting crowdfunding success, little research was conducted to explore the semantic features in this context.

In this study, we addressed this void. We focused on analysis of semantic aspects of project posts to improve the accuracy of funding success predictions. We designed a text analytics framework and developed a prediction model for analyzing and predicting crowdfunding success. We developed a novel model based on semantic features only and achieved similar accuracy level as previous studies. We also developed a prediction model with an impressive F-score of 96.2%, focusing on both project-specific aspects and semantics of project descriptions.

To the best of our knowledge, this study is the first that investigates the relationship between funding success and buzzwords. We show that the *buzzwords* feature is among the features that are highly correlated to funding success compared to both the parameters that we examined and that other researchers examined. We have additionally shown that the features correlated to FS are dependent on project category.

From a practical perspective, the results of our study are highly relevant to fundraisers using crowdfunding web platforms. In addition to the scientific contributions listed above, a set of recommendations that may increase project funding success chances can be proposed. These recommendations are based on the features we listed above, and on influential features from previous studies. We have shown that the category of the project influences the features that have a high correlation with funding success. We offer (example) recommendations for technological projects, as follows:

- Entrepreneurs should update their project information during the funding period.
- The project’s post should contain more feelings words.
- The project’s description should contain buzzwords.

While this paper arrived at very high prediction accuracies of funding success, future research could further improve accuracy by considering the characteristics of images, video content and the semantics of video scripts. Further improvements in the model’s performance could be achieved via novel feature selection algorithms, e.g., [29].
